# *Moringa oleifera* restored semen quality, hormonal profile, and testicular morphology against Highly Active Antiretroviral Therapy-induced toxicity in adult male Wistar rats

**DOI:** 10.5935/1518-0557.20210032

**Published:** 2022

**Authors:** B. Ogunlade, S. O. Jeje, S. A. Adelakun, G.T. Akingbade

**Affiliations:** 1 Human Anatomy Department, Federal University of Technology Akure, Ondo State, Nigeria; 2 Physiology Department, Federal University of Technology Akure, Ondo State, Nigeria

**Keywords:** HAART, *Moringa oleifera*, infertility, sperm parameters, oxidative stress

## Abstract

**Objective:**

Reproductive toxicity has been greatly linked with Highly Active Antiretroviral Therapy (HAART) use. This study investigated the effects of *Moringa oleifera* Leaf Extract (MOE) on HAART-induced testicular toxicity in adult male Wistar rats.

**Methods:**

Twenty adult male Wistar rats (150-200 g) were assigned into four groups (n=5). Group A received distilled water; Group B received (orally) 200 mg/kg BW HAART only; Group C received (orally) 200 mg/kg BW HAART and 100 mg/kg BW MOE (low dose group) and Group D received (orally) 200 mg/kg BW HAART and 300 mg/kg BW MOE. At the end of the 28-day experiment, body and testicular weights were measured; serum and testis obtained were subjected to hormone profiling, biochemical and histological studies.

**Results:**

HAART caused a significant decrease in body and testicular weight, testicular distortion and spermatogenic cell disorganization, altered semen quality and function, hormonal profiles, and oxidative stress markers (SOD, CAT, GSH) were significantly decreased with the concurrent increase in MDA level. However, treatment with MOE improved sperm parameters, testis morphology, antioxidants markers, and hormones assessments.

**Conclusions:**

The exposure to HAART produced marked testicular toxicity, ameliorated using *Moringa oleifera* leaf extract, thereby preserving testicular physiological function and morphology.

## INTRODUCTION

Highly Active Antiretroviral Therapy (HAART) is an active combination regimen capable of relegating Human Immunodeficiency Virus (HIV) to the history books (Granich *et al.* 2010). With the advent of combination regimen (combination antiretroviral therapy - cART), HIV control is reduced via several conditions such as treatment compliance, the toxicity of the drug, and bioavailability (Meynard *et al.,* 2011). There were reductions in the morbidity and mortality rates of associated HIV-infections after the introduction of the HAART regimen in 1996 (Reginald *et al.,* 2011). The consumption of potent cART caused the reconstitution of the immune system, thereby increasing life expectancy and drastically decreasing opportunistic infections (Pineda *et al.,* 2011). However, recent observation of patients discontinuing therapy and relative withdrawal due to significant adverse reactions has prompt researchers to address the treatment failures (Carr & Cooper, 2000). Most clinically reported complications of HAART usage includes AIDS-related insulin resistance, hyperglycemia, gastrointestinal, and lipodystrophy symptoms experienced by several patients (Hagmann, 2003; Montessori *et al.,* 2004). Reproductive organ toxicity associated with HAART usage is increasing globally, especially testicular damage due to differential sensitivity to either the germ or somatic cells and their response to the toxicant (Creasy, 2001; Kushnir & Lewis, 2011). Several manifestations of chemical toxicants have resulted in testicular atrophy with germ cell loss, multinucleated gonocytes, Sertoli cells vacuolization, Leydig cell apoptosis/hyperplasia and ultimately resulting in infertility (Kushnir & Lewis, 2011; Adegoke, 2015). The effects of HAART on a male subject by clinicians are minimal, though studies have substantiated altered sperm qualities among men using the HAART regimen (Bujan *et al.,* 2007).

The exponential increase of medicinal plant use has increased globally over the past decades, despite much unproven scientific validation (Azu *et al.,* 2016). Nearly 21,000 medicinal plants are used globally on different continents (Azu *et al.,* 2016). The most common usage of medicinal plants is against numerous non-communicable diseases like diabetes mellitus as well as infectious diseases like HIV/AIDs (Azu *et al.,* 2016). The originality, relative accessibility, and minimal side effects have increased the use of indigenous plants in recent years, to address the most common ailments around the globe (Mishra *et al.*, 2011). Herbal drugs of medicinal value are now commonly accessible commercially, particularly in developed countries; thereby potentiating their significance in fighting several diseases (Mishra *et al.*, 2011). *Moringa oleifera*, belonging to the family Moringaceae, commonly known as Horseradish in English (Nadkarni, 2009) is one of the best known distributed and naturalized species of the family (Anwar *et al.,* 2007). It is cultivated worldwide (mostly in Asia and Africa), especially in gardens and in house yards, thriving best in rainy or wet weather, and is numerous near the sandy beds of rivers and streams (Anwar *et al.,* 2007). Phytochemical constituents of *Moringa oleifera* leaf extract (MOE) contains specific plant pigments with powerful anti-oxidative potential such as vitamins C, E, A, carotenoids - lutein, kaempferol, quercetin, rutin (Siddhuraju & Becker, 2003; Aslam *et al.,* 2005). Furthermore, the flavonoids present in MOE, such as quercetin and phenolics are responsible for the scavenging of Reactive Oxygen Species (ROS) released from mitochondria, which in turns serve to protect the cells against the deleterious effects of oxidative stress (Kamalakkannan & Prince, 2006; Al-Malki & El Rabey, 2015). In addition, phytochemical constituents of the leaves extracts such as glucosinolates, niazimicin, and benzyl isothiocyanate were reported to be responsible for the anticancer potential of MOE (Nakamura *et al.,* 2002; Hermawan *et al.,* 2012).

MOE has been reported in curing several ailments such as skin infections, asthma, bronchitis, catarrh, and inflammation of the lungs (Mahmood *et al.*, 2010; Hamza, 2010; Singh & Sharma, 2012). In addition, MOE acts against inflammation, hypertension, tumors, free radicals, ulcer, epilepsy, and diabetes (Lai *et al.*, 2010; Paliwal *et al.,* 2011; Sharma *et al.*, 2012; Huang *et al.,* 2012). The response from *Moringa oleifera* leave extracts on HAART-induced alteration in sperm characterization, hormone assessment, antioxidant parameters, and testicular morphology in adult male Wistar rats is investigated.

## MATERIALS AND METHODS

### Chemical

Zidovudine (AZT) 300 mg, Nevirapine (NVP) 200 mg, Lamivudine (3TC) 150 mg (HAART combination therapy) produced by Mylan laboratories Limited, Maharashtra, India was purchased from a commercial source at Matador pharmacy Limited, Akure, Ondo State, Nigeria, from the National Agency for Food Drug Administration and Control (NAFDAC) Reg. NO.: A4-2813 and CAT No: 3061315.

### Plant collection, identification, and Extract preparation

*Moringa Oleifera's f*resh leaves were collected from the Research Farm - Federal University of Technology, Akure, Nigeria, and were identified and authenticated in the herbarium section of the Centre for Research and Development (CERAD) of the Federal University of Technology, Akure, Nigeria. FUTA0190 voucher deposited for reference purposes. The leaves were thoroughly washed and oven-dried at 37^o^C for 48h and pulverized into a smooth powder.

The pulverized sample (850 g) was suspended in 1000 ml of distilled water with regular stirring for 72hrs. The solution obtained was filtered and the resulting filtrate was concentrated in a water bath (40ºC) and yielded 463.21 g of crude extract, corresponding to 58.12% of the residue. The crude extract from *Moringa Oleifera* was kept airtight and refrigerated before use.

### Animals

A total of 20 adult male Wistar rats weighing 150-200 g (aged 8-10 weeks) were obtained from the breeding stock, at the Federal University of Technology, Akure. The rats were collected in isolated cages and acclimatized for 7 days in the experimental house of the Department of Human Anatomy, Federal University of Technology, Akure before the commencement of the experiment. They were maintained with a constant 12h/12h dark and light cycle. The Ethics Committee of the Federal University of Technology, Akure (HREC/07/19/120), approved all animal handling procedures.

### Study design

The rats were divided into four groups (*n*=5), labeled as groups A, B, C, and D. The HAART (Chris-Ozoko *et al.,* 2013) and MOE dosages (Villarruel-López *et al*., 2018) were prepared and administered once daily to the animals. Group A received water. Group B received 200 mg/kg BW HAART (orally) only; Group C received with 200 mg/kg BW HAART (orally) and a low dose of 100 mg/kg BW MOE and Group D received 200 mg/kg BW HAART (orally) (in 2.3 ml of distilled water) and a high dose of 300 mg/kg BW MOE only (in 2.3 ml of distilled water). The experiment lasted for 28 days, after which the animals were slaughtered.

The animals were observed for abnormalities and illnesses. The experimental procedures followed the provided recommendations in the "Guide for the Care and Use of Laboratory Animals" prepared by the National Academy of Sciences. The rats were fed with standard rat chow and water *ad libitum*. The weights of the animals were recorded upon procurement, during acclimatization, at the commencement of the experiment, and weekly throughout the experimental period.

### Surgical procedure

After the completion of the experiment, the animals were administered intraperitoneal pentobarbital sodium (40 mg/kg) and their abdominal regions were dissected and the testes of all the animals were immediately extracted. We collected the serum from the blood samples after centrifuging for analysis. The testicular weights were measured and they were fixed in Bouin's fluid for histological analysis.

### Sperm collection and analyses

The cauda epididymis tissue taken from each animal for sperm analysis was placed in 2 ml of 37ºC salines. The tissue was then crushed by making small incisions and the spermatozoon was dispersed in the saline solution. Sperm count was performed using the Makler counting chamber. Approximately 10µl of sperm suspension samples were taken and evaluated under a light microscope with 10×magnification. The sperm parameters were determined using standard protocols.

### Sperm morphology

This was carried out as described by Saalu *et al.* (2013). The sperm morphology was evaluated with the aid of a light microscope at x400 magnification. The caudal sperm was taken from the original dilution for motility and diluted 1:20 with 10% neutral buffered formalin (Sigma-Aldrich, Canada). We categorized the spermatozoa in wet preparations using phase contrast optics. In this study, a spermatozoon was considered morphologically abnormal if it had a rudimentary tail, round or detached head, and was expressed as a percentage of morphologically normal sperm.

### Biochemical Analysis

The level of lipid peroxidation products was estimated following the method published by Niehaus & Samuelsson (1968). Non-enzymatic antioxidants such as reduced glutathione (GSH) and catalase (CAT) were estimated as described by Ellman (1959) and Sinha (1972), respectively. We determined the SOD activity in the testes according to the method described by Marklund & Marklund (1974).

### Hormone Assessment

Testosterone (TT), Follicle-stimulating hormone (FSH), and Luteinizing hormone (LH) were determined using an immunoassay (ELISA), according to manufacturer instructions.

### Testes Histomorphology

The testes fixed in Bouin's fluid for 24hr and underwent tissue processing technique procedures as described by Adelakun *et al.* (2019); thereafter stained with Hematoxylin and Eosin, and photomicrographs were taken at a magnification of 100x on a Leica DM750 microscope.

### Data presentation and statistical analysis

We analyzed the data statistically with one-way ANOVA, followed by Dunnett's comparison test using GraphPad Prism 5 Windows (GraphPad Software, San Diego, California, USA). Values expressed as Mean±SEM. The level of significance was *p*<0.05.

## RESULTS

### Body and testicular weights

#### Effects of MOLE on the body and testicular weights on normal and HAART-treated rats

**Figure 1:**
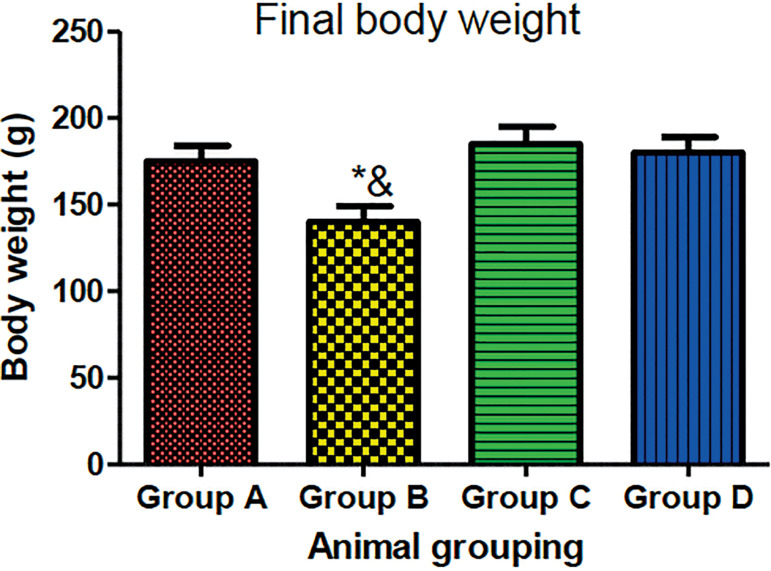
Effect of MOE on body weight on HAART-induced normal and experimental rats. *p<0.05 as compared to group A; & *p*<0.05 as compared to groups C and D.

**Figure 2:**
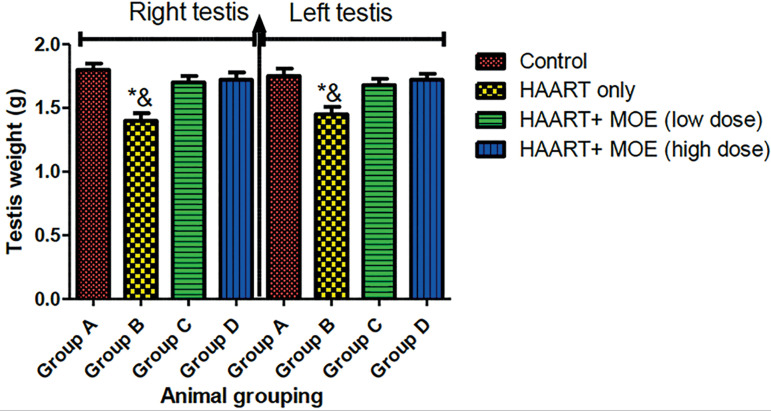
Effect of MOE on testes weight (right and left) on HAART-induced normal and experimental rats. *p<0.05 as compared to group A; & *p*<0.05 as compared to groups C and D.

The result revealed that rats treated with HAART showed a significant decrease in body and testicular weights relative to control animals (*p*<0.05) (Figs. 1 and 2). However, the rats administered with both HAART and MOLE in high and low doses showed a significant increase in their final body weight and testicular weight relative to the HAART-only group (*p*<0.05) (Figs. 1 and 2).

### Semen characterization

#### Effects of MOE on sperm morphology (neck, tail, and head defects and normal) on normal and HAART-treated rats

**Figure 3:**
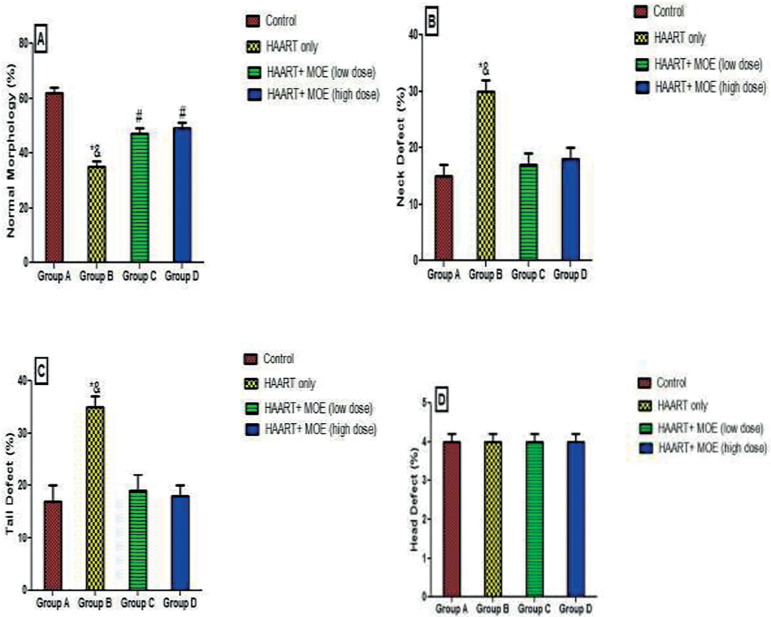
Effect of MOE on sperm morphology (Neck, Tail, and Head defects and Normal) on HAART-induced normal and experimental rats. *p<0.05 as compared to group A; & *p*<0.05 as compared to groups C and D.

The results revealed that rats treated with HAART only showed a significant decrease (*p*<0.05) in morphology (normal) with a concomitant increase in neck, tail, and head defects, relative to the controls (Fig. 3). However, the rats administered with both HAART and MOE in high and low doses had a significant increase in morphology (normal), with a concomitant decrease in neck, tail, and head defects when compared with the HAART-only group (*p*<0.05; Fig. 3).

**Figure 4:**
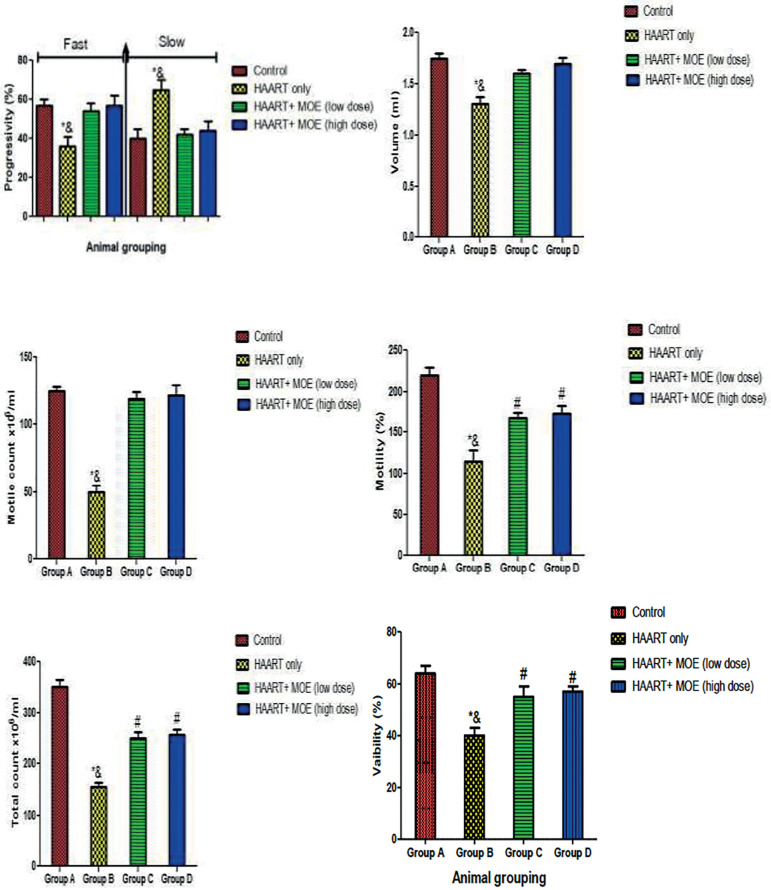
Effect of MOE on Progressive assessment (fast and slow) on HAART-induced normal and experimental rats. *p<0.05 as compared to group A; & *p*<0.05 as compared to groups C and D.

Furthermore, the results showed that rats treated with HAART-only showed a significant decrease (*p*<0.05) in progressive assessment (fast), volume, motile count, percentage motility, total count, and viability with a concomitant increase in progressive assessment (slow), relative to the control animals (Fig. 4). However, the rats administered with both HAART and MOE in high and low doses had a significant increase in progressive assessment (fast), volume, motile count, percentage motility, total count, and viability with a concomitant decrease in progressive assessment (slow) relative to the HAART-only group (*p*<0.05)(Fig 4).

### Biochemical assay

#### Effects of MOE on MDA, GSH, SOD, and CAT level on HAART-treated and normal rats

**Figure 5:**
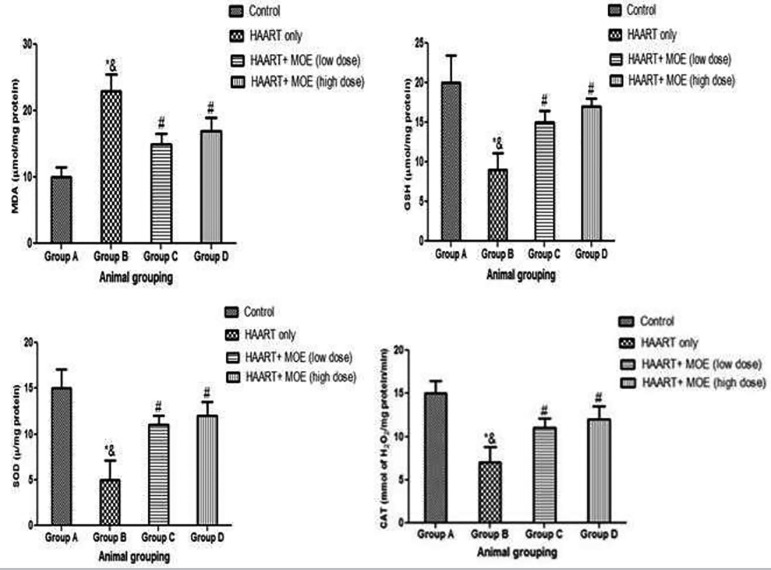
Effect of MOE on MDA, GSH, SOD, and CAT levels on HAART induced normal and experimental rats. *p<0.05 as compared to group A; & *p*<0.05 as compared to group C and D; #p<0.05 as compared to groups A.

The results showed that rats treated with HAART only showed a significant decrease in GSH, SOD, and CAT activities (Fig. 5) with a corresponding increase in MDA relative to the control animals (*p*<0.05; Fig. 5). However, the rats administered with both MOE and HAART in high and low doses showed a significant increase in GSH, SOD and, CAT activities (Fig. 5) with the corresponding decrease in MDA relative to the HAART-only group, (Fig. 5) except in MDA, which showed a significant decrease (*p*<0.05; Fig. 5). Although, there was a significant decrease in GSH, SOD, and CAT activities when the rats administered both MOE and HAART in high and low doses relative to the control, (Fig. 5) except in MDA, which shows a significant increase (*p*<0.05; Fig. 5).

### Hormonal assay

#### Effects of MOE on serum level of FSH, LH, and TT on HAART-treated and normal rats

**Figure 6:**
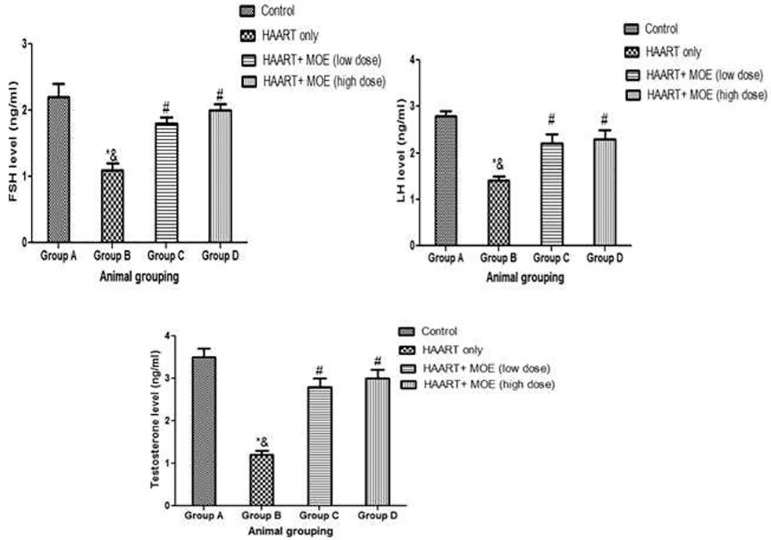
Effect of MOE on serum level of FSH, LH and TT on HAART-induced normal and experimental rats. *p<0.05 as compared to group A; & *p*<0.05 as compared to group C and D; #*p*<0.05 as compared to group A.

The results revealed that rats treated with HAART only showed a significant decrease in FSH, LH, and TT activities relative to the control animals (*p*<0.05; Fig. 6). However, the rats administered with both MOE and HAART in high and low doses showed a significant increase in FSH, LH, and TT activities relative to the HAART-only group (*p*<0.05; Fig. 6). Although, there was a significant decrease in FSH, LH, and TT activities when the rats administered both MOE and HAART in high and low doses relative to the control animals (*p*<0.05; Fig. 6).

### Histological evaluations

**Figure 7:**
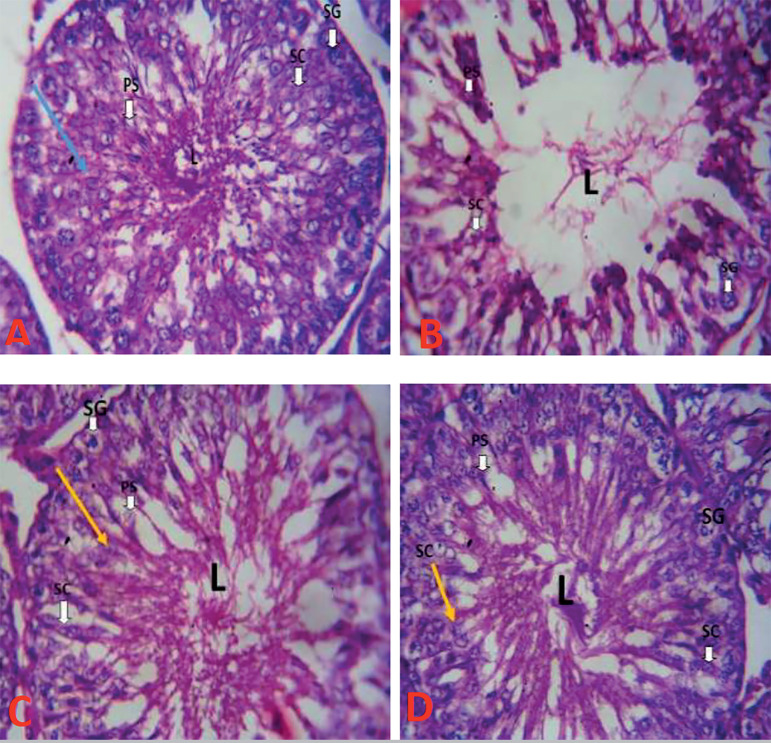
Testicular Photomicrographs showing the effects of MOE on HAART induced normal and experimental rats. Scale bar: 250µm. Stains: Hematoxylin and Eosin, Magnification X400. A: Group A showing testicular photomicrograph section of control rat with normal histoarchitecture of typically organized layers of Sertoli cells (arrow), no pathological changes in the lumen (L) of the seminiferous tubules, with prominent spermatogonia (SG), Primary spermatocyte (PS). B: Group B showing testicular photomicrograph section of HAART only group with distortion in the tubular architecture and disorganization of the Sertoli cells within the seminiferous tubules, hypocellularity of spermatogonia, disruption of spermatogenesis and empty lumen (L). C: Group C showing testicular photomicrograph section of MOE and HAART treated group at low dose with visible restored lumen (L), spermatogonia (SG), Primary spermatid (PS) and abundant Sertoli cell (yellow arrow). D: Group D showing testicular photomicrograph section of MOE and HAART treated group at high dose showing visible lumen (L), spermatogonia (SG), Primary spermatid (PS) and abundant Sertoli cell (yellow arrow).

The testicular histoarchitecture of the HAART-only group (B) showed distortions in the tubular structure and disorganization of the Sertoli cells within the seminiferous tubules, hypocellularity of spermatogonia, disruption of spermatogenesis, empty lumen, decrease in germinal epithelium thickness, and reduction in the diameter of the seminiferous tubules when compared with the controls (Group A; Fig. 7). In addition, HAART distorted the seminiferous tubules with loss of normal distribution of epithelial lining and vacuolar cytoplasm compared with the controls (Group A). However, testicular photomicrograph of the control group had similar characteristics with the high and low dose MOE rats (groups C and D), showing oval or circular presentation with distinctive stratified seminiferous epithelium with lumens possessing spermatogenic cells and prominent Leydig cells (Fig. 7). The testicular section of the rats (groups C and D) administered with both MOE and HAART at high and low doses, respectively, showed restored microarchitecture of the testicular morphology with mild distortion of the tubular architecture and disorganization of the spermatogenic cells in the seminiferous tubules (Fig. 7).

## DISCUSSION

In this study, the combination HAART therapy (Zidovudine, Lamivudine, and Nevirapine) causes significant changes in the body and testicular weights. Previous observations showed that spermatogenesis is capable of causing declines in testicular structures, which is evident in our study. Additionally, the weight coefficient (testicular and body weight ratio) is an important sensitive and effective prediction of chemical toxicity (Asagba *et al.,* 2007). The significant decrease in the weight coefficient of testicular tissue and final body weight in HAART-only treated animals (Group B) compared to controls further corroborate HAART toxicity. Gonadotoxicity occurs through organ swelling, atrophy, or hypertrophy (Afolayan & Yakubu, 2009). The final body and testicular weights observed in this study corroborated the report by Azu (2012) that revealed that HAART exposure in rats causes shrinkage of the nuclear size of testicular cells and declines in body weight. However, the combination treatment of HAART and MOE at low and high doses (groups C and D respectively), preserves the body and testicular weights associated with HAART toxicity in a dose-dependent manner, thereby mopping up excessive free radicals associated with organ atrophy, which could be attributed to the powerful phytonutrients present within the plants responsible for the preservation of the body and testicular weights, such as vitamins C, E, A; kaempferol, quercetin, rutin (Siddhuraju & Becker, 2003; Aslam *et al.*, 2005; Kamalakkannan & Prince, 2006; Al-Malki & El Rabey, 2015).

The sperm morphology in HAART-treated animals showed a significant defect in the neck and tail regions. This finding conforms to the report by Nicopoullos *et al.* (2010) that showed testicular morphological derangement as a potential target for HAART, thereby revealing that HAART rather than HIV-1, was responsible for the topographic changes in sperm cells. However, the combined administration of HAART and MOE at low and high doses ameliorates the toxicity brought about by HAART exposure in a dose-dependent mechanism.

Furthermore, our study found a significant decrease in the progressive assessment (slow movement), decreased sperm motility, decrease in semen volume, and decrease in total sperm count in HAART-treated animals. Our findings are following previous reports that HIV-1 infection and/or treatment with HAART creates distortions in semen volume and cellular composition (Sertoli or spermatogenic cells), decreased sperm motility, decrease in rapidly progressive motile spermatozoa movement, and decrease in sperm concentration, total count and progressive motility (Azu *et al.,* 2014). However, treatment with MOE at low and high doses mitigated the toxicity brought on by HAART exposure in the semen parameters in a dose-dependent manner; thereby suggesting the antioxidant and protective role of MOE against chemical toxicants. The ameliorative potential exhibited by MOE could be attributed to the radical scavenging activities and the distinct and significant diversity of phytochemicals reacting uniquely with various free radicals (Kim *et al.,* 2003). Previous studies revealed that MOE possesses significant antioxidant activities attributed to a variety of compounds such as anthocyanins and reductones (Custódio *et al*., 2013).

Furthermore, there was a significant increase in MDA levels, with a concomitant decrease in GSH, CAT, and SOD activities in HAART-treated animals, hence, proving that the testicular tissue of the untreated rats is greatly affected by ROS due to chemically-induced oxidative stress. Our study supports previous findings that revealed an increase in testicular MDA levels after the administration of HAART drugs (Oputiri & Elias, 2014; Adaramoye *et al.,* 2015). Additionally, the previous study revealed that HAART significantly reduced testicular GSH, SOD, and CAT, consequently reducing antioxidant availability; thereby increasing free radical activity and disrupting the redox balance (Ogedengbe *et al.,* 2018). It has also been reported that an imbalance in redox activity, due to oxidative stress, is highly deleterious to tissue functionality (Rahman *et al.,* 2012; Oyeyipo *et al.,* 2018). However, co-treatment of HAART with MOLE at low and high doses significantly restored lipid peroxidation and raised the levels of GSH, CAT, and SOD in a dose-dependent mechanism, thereby revealing the potential of MOE acting as an antioxidant in mopping up free radicals and ROS after chemical insults. Additionally, the observed restoration of the antioxidant markers could be attributed to the abundant polyphenols present in MOE, which have the electron donor potency capable of detoxifying lipid peroxidation and antioxidant enzyme activation (Jaiswal *et al*., 2009; Alvarez-Suarez *et al*., 2011; Oboh *et al*., 2020).

Also, a significant decrease in FSH, LH and TT levels in HAART treated animals was recorded, which agrees with previous studies that showed that HAART causes deterioration in the functionality of the Sertoli and Leydig cells, weakening spermatogenesis due to decreased FSH, LH, and TT levels (Kiyanifard *et al.,* 2010; Rahman *et al.*, 2012; Ogedengbe *et al.,* 2018). However, co-administration of HAART and MOE at low and high doses restored the hormone profiles by elevating the FSH, LH, and TT levels in a dose-dependent mechanism; thereby maintaining the physiological function of the testis. Hormonal assessments are an important factor in testicular homeostasis involving the gonadotropin-releasing hormone from the hypothalamus, follicle-stimulating hormone; and luteinizing hormone. The previous report deduced that both luteinizing hormone and follicle-stimulating hormone are necessary for spermatogenesis (Ogedengbe *et al.,* 2018), while follicle-stimulating hormone (FSH) is regarded as a biological marker for accessing Sertoli cell functionality in the spermatogenic process (Ogedengbe *et al.,* 2018). Furthermore, histopathological features revealed significant degeneration of spermatogenic cells with thinning and disorganization of the basement membrane, widened lumen, and hypocellular interstitium observed in the HAART- only group (Group B), compared with the control. This result conforms to previous studies that revealed HAART capability in compromising the morphological integrity of the testicular epithelium and blood-testis barrier of the Sertoli cells (Vasilaki & McMillan, 2011; Ogedengbe *et al.,* 2018). However, these structural changes were mitigated after the administration of MOE at low and high doses, thereby restoring the lumen of the seminiferous tubules with visible spermatozoa and abundant sperm cells. Previous assessment of MOE showed that the polyphenolic compounds and various antioxidant activities are responsible for the positive restoration of testicular histomorphology, indicating that polyphenols may be due to their hydroxyl groups, thereby serving as major contributors to the free radical scavenging ability of the extracts (Chew *et al.,* 2009).

## CONCLUSION

This study ascribed that HAART reduced body and testicular weights, caused changes to hormone profiles, oxidative stress parameters, and distinctive distortion of the testicular morphology and disorganization of the spermatogenic cells. However, *Moringa oleifera* leaves had a protective response against HAART toxicity, attributed to the presence of potent phytochemical compounds (such as kaempferol, quercetin, niazimicin, and benzyl isothiocyanate) capable of preserving testicular morphology and functions.
